# Management and clinical outcome of stable coronary artery disease in Austria: Results from 5 years of the CLARIFY registry

**DOI:** 10.1007/s00508-017-1248-1

**Published:** 2017-09-14

**Authors:** Irene M. Lang, Roza Badr-Eslam, Nicola Greenlaw, Robin Young, Philippe Gabriel Steg

**Affiliations:** 1 Department of Cardiology, AKH-Vienna, Medical University of Vienna, Vienna, Austria; 2 Robertson Center of Biostatistics, University of Glasgow, Glasgow, UK; 3 Département Hospitalo-Universitaire FIRE, Hôpital Bichat, Assistance Publique – Hôpitaux de Paris, Paris, France; 4 NHLI Imperial College, ICMS, Royal Brompton Hospital, London, UK

**Keywords:** Coronary artery disease, CLARIFY, Treatment, Clinical outcome, Austria

## Abstract

**Background:**

The population of patients with established coronary artery disease (CAD) is growing because of an improvement in outcomes and survival from acute disease episodes. Nevertheless, these patients remain at high risk of cardiovascular events. Thus, CAD management is important in prevention of disease progression. The objective of this analysis was to describe disease management and clinical outcome of Austrian outpatients with stable CAD over 5 years by using data from the international CLARIFY registry.

**Methods:**

CLARIFY was an international prospective observational registry of outpatients with stable CAD, defined as prior myocardial infarction or revascularization (CABG or PCI), coronary stenosis of more than 50% by coronary angiography or chest pain with myocardial ischemia. We analyzed demographic characteristics, risk factors, treatments and clinical outcomes of 424 Austrian outpatients with established CAD who were enrolled between November 2009 and July 2010 and observed until September 2015.

**Results:**

The primary risk factors in Austrian outpatients with stable CAD were smoking (current smokers: 13.2%), overweight (77.1%), hypertension (78.5%), raised low-density lipoprotein (LDL) cholesterol plasma levels (81.4% ≥ 0.7 g/l or 1.8 mmol/l), elevated heart rate (≥70 bpm: 60.9% in patients with anginal symptoms) and poor physical activity (none or light activity: 63.4%). Patients received lipid-lowering drugs (predominantly statins), aspirin, beta-blockers and angiotensin-converting enzyme (ACE) inhibitors according to current recommendations. After 5 years a systolic blood pressure (SBP) < 140 mm Hg and diastolic blood pressure (DBP) < 90 mm Hg was reached in 58.5% of patients. Of the patients 70.4% had LDL cholesterol plasma levels below 1.0 g/l (2.6 mmol/l), 42.1% of smokers had stopped smoking, 42.9% of patients with anginal symptoms had a heart rate ≤60 bpm and 26.0% of diabetic patients had brought their HbA1c levels below 6.5%. Cardiovascular death, myocardial infarction or stroke occurred in 30 patients (7.1%), all-cause death in 25 cases (5.9%) and cardiovascular death in 15 cases (3.5%). Myocardial infarction was reported in 14 patients (fatal and non-fatal: 3.3%) and stroke in 8 patients (fatal and non-fatal: 1.9%), 39 patients (9.2%) underwent myocardial revascularization and 124 patients (29.2%) experienced cardiovascular hospitalization.

**Conclusion:**

Characteristics of Austrian outpatients with stable CAD corresponded to those of patients with CAD in other developed countries. Medical treatments following the recommendations of the European guidelines were prescribed in the majority of patients; however, recommended goals of life style interventions including a heart rate less than 60 bpm and general risk factor management were not achieved by a high proportion of patients. Heart rate control and life style changes remain unmet needs of cardiovascular care in Austria.

## Introduction

Approximately 17.5 million people die each year from cardiovascular disease. An estimated 30% of all deaths worldwide are due to heart attacks and strokes. Coronary artery disease (CAD) accounts for the greatest proportion of cardiovascular diseases. Overall, CAD is the leading cause of death worldwide and is predicted to remain so for the next 20 years [1–3]. In Europe between 14% and 20% of women and 16–25% of men die from CAD [4]. Furthermore, CAD is a leading cause of morbidity and poor quality of life. Thus, CAD is a major public health problem which exerts heavy economic costs. Overall, CAD is estimated to have cost the European Union €45 billion in 2003 and this estimate even might be an underestimate [5]. Nevertheless, improved management of the disease consisting of life style interventions, revascularization or optimal drug therapy have resulted in a decrease in CAD mortality since 1995 by 34% [6, 7]; however, most data regarding CAD are from patients with acute coronary symptoms or in the case of patients with stable CAD come from randomized trials of drugs used in the prevention of cardiovascular events (e. g. EUROPA [8]). In order to generalize the findings from these trials, real-life aspects regarding demography and disease management of outpatients with stable CAD are needed. Because of the improvement in outcomes and survival from episodes of acute disease, the population of patients with established CAD is growing. All these patients remain at high risk of cardiovascular events and therefore disease management is important in prevention of disease progression [9, 10].

The prospeCtive observational LongitudinAl RegIstry oF patients with stable coronary arterY disease (CLARIFY) registry was initiated to improve knowledge regarding patients with stable CAD. The registry was designed to collect data on the current status of outpatients with stable CAD, including demography, clinical profiles, therapeutic strategies and clinical outcomes. In this context, the registry includes symptomatic and asymptomatic patients. The main objectives were to define contemporary patients with stable CAD, to identify discrepancies between evidence-based recommendations and practice, and to investigate long-term prognostic determinants [10, 11].

This analysis describes disease management and clinical outcomes of Austrian outpatients with stable CAD over 5 years of CLARIFY. Success in disease management and treatment were evaluated by analysis of patients who achieved the following treatment targets: normalized blood pressure, lowered low-density lipoprotein (LDL) cholesterol levels, heart rate ≤60 bpm in patients with angina, smoking cessation, and lowered glycated hemoglobin (HbA1c) levels in diabetic patients. In addition, the analysis included a comparison of heart rate and usage of heart rate-modulating medications of Austrian CAD patients with European CAD patients.

## Materials and methods

### Study design and patients

CLARIFY was an international, prospective, observational, longitudinal registry in stable CAD outpatients with 5 years of follow-up. The study rationale, design and methods were described previously [10, 12]. Overall, patients were enrolled in 45 countries worldwide. They were treated according to usual clinical practice at each institution, with no specific tests or therapies defined in the study protocol.

Patients were enrolled when they met at least one of the following inclusion criteria: documented myocardial infarction (more than 3 months ago), coronary stenosis of more than 50% proven by coronary angiography, chest pain with myocardial ischemia proven by stress electrocardiogram, stress echocardiography or myocardial imaging, and coronary artery bypass grafting (CABG) surgery or percutaneous coronary intervention (PCI) more than 3 months ago. Patients were excluded in cases of hospitalization for cardiovascular disease within the past 3 months (including revascularization) and planned revascularization as well as patients with conditions expected to hamper participation or the 5‑year follow-up, such as limited cooperation, limited legal capacity, serious non-cardiovascular disease or conditions interfering with life expectancy (e. g. cancer, drug abuse) or severe cardiovascular disease (e. g. advanced heart failure, severe valve disease, history of valve repair and/or valve replacement).

To ensure that the study population was representative of a real-life population of stable CAD outpatients in each country, recruitment was based on a predefined selection of physician types (cardiologists, internists, primary care physicians) and aimed for consecutive enrolment of eligible patients. Each physician recruited a maximum of 15 outpatients with stable CAD as defined by the inclusion criteria, the general target enrolment for any participating country was 25 patients per million inhabitants (range 12.5–50). The first patient was included on 26 November 2009 and recruitment was completed on 30 June 2010. The registry ended in September 2015. A total of 424 Austrian patients were included in the registry, 28.1% of patients were lost to follow-up after 5 years. The study was registered in the ISRCTN registry of clinical trials (ISRCTN43070564).

### Data collection and data management

Investigators completed electronic case report forms (eCRFs) at baseline and at patient visits 1 year, 2 years, 3 years, 4 years and 5 years after enrolment (±3 months). Data collected at baseline included demographics, risk factors and life style, medical history and current symptoms, physical examination including heart rate determined by pulse palpation and the results of the most recent electrocardiogram (ECG) performed within the past 6 months, laboratory values (if available: fasting blood glucose, HbA1c, cholesterol, including total cholesterol, high-density lipoprotein cholesterol (HDL), LDL, triglycerides, plasma creatinine, and hemoglobin), and current chronic medical treatments taken by the patient for more than 7 days prior to inclusion in the registry. During annual follow-up visits the following information was collected: employment status, risk factors and life style, current symptoms, physical examination including heart rate, laboratory values (if available), current treatments and clinical events occurring since the last visit, e.g. all-cause death, fatal or non-fatal myocardial infarction (MI), fatal or non-fatal stroke, cardiovascular death, unstable angina, major bleeding, coronary angiography, and revascularization percutaneous coronary intervention (PCI) and CABG, transient ischemic attack (TIA), hospitalization for congestive heart failure (CHF) and cardiovascular hospitalization. Completed eCRFs were electronically sent to the data management center in Glasgow (Robertson Centre for Biostatistics, University of Glasgow, UK) where checks for completeness, internal consistency and accuracy were run.

The study was performed in accordance with the principle of the Declaration of Helsinki and was approved by the National Research Ethics Service, Isle of Wright, Portsmouth and Southeast Hampshire Research Ethics Committee, UK. Approval was also obtained in Austria, in accordance with local regulations before recruitment of the first participant. All patients signed a written informed consent to participate, in accordance with national and local guidelines.

### Statistical analysis

All data were collected and analyzed at an independent academic statistic center at the Robertson Centre for Biostatistics, University of Glasgow, UK which was responsible for data management, statistical analyses and storage of the data according to regulations. The main analyses were designed to describe patient characteristics at baseline and to estimate the annual cardiovascular event rates, and the outcome event rates by region and country. Baseline variables as well as parameters collected in the annual follow-up visits were summarized as means, standard deviations (SD), medians, interquartile ranges (IQR), and ranges for continuous data and as counts and percentages for categorical data. Summaries were provided for CLARIFY Austria and for CLARIFY Austria and subjects with angina. Statistical analysis was performed using the statistical package R, version 3.1.2 [13]. Height and weight were used to calculate body mass index (BMI) in kg/m^2^. Outcome was defined as occurrence of death and/or any cardiovascular events, such as MI or stroke during the observation period. Treatment targets were defined as normalized blood pressure (<140/90 mm Hg), lowered LDL cholesterol plasma levels (<0.7 g/l or <1.0 g/l), lowered HbA1c levels in diabetic patients (<6.5% or <7.0%), heart rate ≤60 bpm in patients with anginal symptoms and smoking cessation.

## Results

In Austria, 424 patients with stable CAD were included in the CLARIFY registry of which 75.0% were male and the mean age was 65.2 ± 10.1 years. The most frequent concomitant diseases were dyslipidemia and treated hypertension, 53.3% had a medical history of MI and 77.6% had had a PCI in the past and 33.5% of patients had a family history of premature CAD. Symptoms of chronic heart failure were present in 12.3%. At baseline, mean BMI was 27.7 ± 3.9 kg/m^2^ and mean blood pressure was within the normal range. Resting heart rate measured by pulse palpation was 68.3 ± 10.3 bpm and by ECG 67.2 ± 10.8 bpm, 57.1% of patients were former or current smokers. The most predominant CAD risk factors were overweight and elevated LDL cholesterol plasma levels. Most patients reported regular light physical activities (52.8%), 46 patients (10.8%) with stable CAD were affected by anginal symptoms and 65.2% of these patients were male but the proportion of patients with anginal symptoms was higher in women than in men (15.1% in women vs. 9.4% in men). Angina was classified as grade II according to the Canadian Cardiovascular Society (CCS) classification in most cases. Mean age of patients with anginal symptoms was 68.6 ± 9.2 years and dyslipidemia was the most frequently documented concomitant disease. Compared with the complete Austrian patient population, previous PCI was less common in patients with angina but symptoms of chronic heart failure were more common after previous PCI. In addition, patients with angina showed a slightly higher mean BMI and higher mean heart rate. Overall, in 60.9% of patients with angina heart rate was ≥70 bpm. The most common risk factors were elevated LDL cholesterol plasma levels and overweight. In addition, 32.6% of patients with angina were former smokers and 28.3% current smokers (Table 1).

**Table 1:** Baseline characteristics Austria^n^

Parameter	Patients with stable CAD (*N* = 424)	Patients with stable CAD and angina (*N* = 46)
Age (mean years ± SD)	65.2 (10.1)	68.6 (9.2)
Gender (*N* [%])		
Male	318 (75.0)	30 (65.2)
Female	106 (25.0)	16 (34.8)
Employment		
Employed full-time	94 (22.2)	3 (6.5)
Employed half-time	11 (2.6)	2 (4.3)
Unable to work	3 (0.7)	0 (0.0)
Unemployed	4 (0.9)	2 (4.3)
Retired	310 (73.1)	38 (82.6)
Other	2 (0.5)	1 (2.2)
Education		
Primary school (or less)	214 (50.5)	29 (63.0)
Secondary school	169 (39.9)	16 (34.8)
College/University	41 (9.7)	1 (2.2)
*Concomitant diseases and lifestyle (N [%])*		
Family history of premature CAD^a^	142 (33.5)	14 (30.4)
Treated hypertension	333 (78.5)	40 (87.0)
Diabetes	121 (28.5)	19 (41.3)
Dyslipidemia^b^	375 (88.4)	42 (91.3)
PAD	64 (15.1)	11 (23.9)
Alcohol intake, glasses per week		
0	99 (23.3)	14 (30.4)
>0 to <20	316 (74.5)	31 (67.4)
>20	9 (2.1)	1 (2.2)
Stimulant drinks		
Coffee	311 (73.3)	37 (80.4)
Tea	29 (6.8)	6 (13.0)
Neither	84 (19.8)	3 (6.5)
Physical activity		
None	45 (10.6)	6 (13.0)
Light^c^	224 (52.8)	32 (69.6)
1–2 times per week^d^	81 (19.1)	7 (15.2)
≥3 times per week^d^	74 (17.5)	1 (2.2)
*Medical history N (%)*		
Myocardial infarction	226 (53.3)	21 (45.7)
PCI	329 (77.6)	31 (67.4)
CABG	99 (23.3)	12 (26.1)
Abdominal aortic aneurysm	10 (2.4)	1 (2.2)
Coronary stenosis^e^	54 (12.7)	14 (30.4)
ICD	6 (1.4)	0 (0.0)
Pacemaker	7 (1.7)	1 (2.2)
Stroke	17 (4.0)	5 (10.9)
TIA	13 (3.1)	3 (6.5)
Hospitalization for CHF	13 (3.1)	2 (4.3)
Atrial fibrillation/flutter	39 (9.2)	4 (8.7)
Asthma/COPD	52 (12.3)	12 (26.1)
*Current symptoms (N [%])*		
Angina	46 (10.8)	46 (100.0)
CCS class (if angina)		
I	14 (30.4)	14 (30.4)
II	25 (54.3)	25 (54.3)
III	7 (15.2)	7 (15.2)
CHF symptoms	52 (12.3)	15 (32.6)
NYHA class (if CHF)		
II	45 (86.5)	13 (86.7)
III	7 (13.5)	2 (13.3)
*Measurements*		
Weight [kg]	81.4	81.0
Height [cm]	171.5	170.0
BMI, mean (SD) [kg/m^2^]	27.7 (3.9)	28.0 (4.9)
Heart rate (by pulse palpation), mean (SD) [bpm]	68.3 (10.3)	73.8 (13.8)
Heart rate (by pulse ECG), mean (SD) [bpm]	67.2 (10.8)	72.0 (14.3)
Heart rate ≥70 bpm (in angina patients)	–	28 (60.9)
SBP, mean (SD), [mm Hg]	133.8 (16.6)	135.3 (18.2)
DBP mean (SD), [mm Hg]	80.0 (9.1)	80.4 (10.7)
Waist circumference, mean (SD), [cm]	99.2 (11.2)	101.5 (13.9)
*Risk factors (N (%))*		
Overweight f	327/424 (77.1)	34/46 (73.9)
Obesity^g^	99/424 (23.3)	15/46 (32.6)
Smoking status, N	424	46
Current	*56 (13.2)*	*13 (28.3)*
Former	*186 (43.9)*	*15 (32.6)*
Never	*182 (42.9)*	*18 (39.1)*
Raised blood pressure^h^	175/424 (41.3)	23/46 (50.0)
Raised LDL cholesterol 1^i^	126/370 (34.1)	12/39 (30.8)
Raised LDL cholesterol 2^j^	301/370 (81.4)	31/39 (79.5)
Lowered HDL cholesterol^k^	66/379 (17.4)	12/42 (28.6)
Raised HbA1c^l^	46/121 (38.0)	5/19 (26.3)
HR on palpation ≥70 bpm^m^	–	28/46 (60.9)

*BMI* body mass index, *bpm* beats per minute, *CABG* coronary artery bypass graft, *CAD* coronary artery disease, *CCS* Canadian Cardiovascular Society, *CHF* chronic heart failure, *COPD* chronic obstructive pulmonary disease, *DBP* diastolic blood pressure, *ECG* electrocardiogram, *HbA1c* glycated hemoglobin, *HDL* high-density lipoprotein, *HR* heart rate, *ICD* internal cardiac defibrillator, *LDL* low-density lipoprotein, *N* number of patients, *NYHA* New York Heart Association, *PAD* peripheral artery disease, *PCI* percutaneous coronary intervention, *SBP* systolic blood pressure, *SD* standard deviation, *TIA* transient ischaemic attack

^a^Myocardial infarction, sudden death, stable angina at age <55 years (men) or <65 year (women) in a first-degree relative

^b^Defined as history of documented total cholesterol >2 g/l or >5.18 mmol/l or high-density lipoprotein cholesterol <0.4 g/l or <1 mmol/l

^c^Light physical activity most weeks

^d^At least 20 min vigorous physical activity

^e^Coronary territories with stenosis >50% at coronary angiography or required revascularization in medical history

^f^Defined as BMI ≥25–29.99 kg/m^2^

^g^Defined as BMI ≥30 kg/m^2^

^h^Defined as systolic blood pressure ≥140 mm Hg and diastolic blood pressure ≥90 mm Hg

^i^Defined as LDL cholesterol plasma level ≥1 g/l or 2.6 mmol/l

^j^Defined as LDL cholesterol plasma level ≥0.7 g/l or 1.8 mmol/l

^k^Defined as HDL cholesterol plasma level ≤40 mg/dl or 1.0 mmol/l

^l^in diabetic patients, defined as HbA1c level ≥7.0%

^m^only in angina patients

^n^mean values as well as percentages are of the data available

At baseline, the majority of patients were treated with lipid-lowering drugs (LLD) (94.6%, predominantly statins: 89.3%), aspirin (86.6%) and beta-blockers (78.8%). Angiotensin-converting enzyme (ACE) inhibitors were used in 45.0% and 33.0% received diuretics. Trimetazidine and ranolazine were not used. The most frequently received other medications were proton pump inhibitors (PPI) and antidiabetic agents. During the 5 years of CLARIFY, there were only few alterations to these treatments and LLD (predominantly statins), aspirin and beta-blockers were the primary medications on follow-up. The usage of LLD was relatively stable, whereas the proportion of patients who received aspirin, beta-blockers or ACE inhibitors decreased over time. In contrast, the use of diuretics and angiotensin II (AT II) receptor inhibitors increased over time. With respect to other medications the proportion of patients who received non-steroidal anti-inflammatory drugs (NSAIDs) slightly decreased. All other treatments remained the same (Table 2).

**Table 2:** Treatment of Austrian patients with stable CAD and Austrian patients with stable CAD and angina during observation (*N* [%])^a^

Parameter	Baseline	1st year	2nd year	3rd year	4th year	5th year
*Patients with stable CAD (N [%])*						
*N*	*424*	*411*	*351*	*337*	*323*	*305*
Aspirin	367 (86.6)	333 (82.2)	268 (79.1)	248 (75.8)	231 (74.5)	213 (73.2)
Thienopyridines	38 (9.0)	42 (10.4)	35 (10.3)	34 (10.4)	34 (11.0)	30 (10.3)
Other antiplatelet agent	44 (10.4)	39 (9.6)	31 (9.1)	34 (10.4)	32 (10.3)	33 (11.3)
Oral anticoagulant	46 (10.8)	44 (10.9)	45 (13.3)	45 (13.8)	48 (15.5)	46 (15.8)
Beta-blocker	334 (78.8)	304 (75.1)	249 (73.5)	235 (71.9)	223 (71.9)	202 (69.4)
Ivabradine	25 (5.9)	26 (6.4)	18 (5.3)	17 (5.2)	15 (4.8)	16 (5.5)
Calcium antagonists	76 (17.9)	74 (18.3)	60 (17.7)	63 (19.3)	55 (17.7)	52 (17.9)
Verapamil or dilitiazem	15 (3.5)	15 (3.7)	11 (3.2)	10 (3.1)	8 (2.6)	6 (2.1)
ACE inhibitors	191 (45.0)	175 (43.2)	145 (42.8)	134 (41.0)	122 (39.4)	117 (40.2)
AT II receptor-blocker	120 (28.4)	120 (29.6)	105 (31.0)	100 (30.6)	97 (31.3)	89 (30.6)
LLD	401 (94.6)	378 (93.3)	316 (93.2)	300 (91.7)	284 (91.6)	267 (91.8)
Statins (if LLD)	358 (89.3)	321 (84.9)	261 (82.6)	246 (82.0)	230 (81.0)	213 (79.8)
Other antianginal agent	50 (11.8)	50 (12.3)	38 (11.2)	40 (12.2)	37 (11.9)	32 (11.0)
Trimetazine	0 (0.0)	0 (0.0)	0 (0.0)	0 (0.0)	0 (0.0)	0 (0.0)
Ranolazine	0 (0.0)	1 (0.2)	2 (0.6)	4 (1.2)	4 (1.3)	3 (1.0)
Diuretics	140 (33.0)	135 (33.3)	119 (35.1)	111 (33.9)	111 (35.8)	105 (36.1)
Other hypertensive agent	47 (11.1)	50 (12.3)	39 (11.5)	36 (11.0)	34 (11.0)	29 (10.0)
Digoxin and derivates	10 (2.4)	9 (2.2)	8 (2.4)	7 (2.1)	7 (2.3)	9 (3.1)
Amiodarone/dronedarone	17 (4.0)	16 (4.0)	13 (3.8)	13 (4.0)	12 (3.9)	11 (3.8)
Other antiarrhythmics	8 (1.9)	8 (2.0)	10 (2.9)	8 (2.4)	8 (2.6)	7 (2.4)
Other medication						
NSAIDs	44 (10.4)	44 (10.9)	35 (10.3)	28 (8.6)	27 (8.7)	22 (7.6)
Antidiabetic agents	82 (19.3)	76 (18.8)	56 (16.5)	56 (17.2)	54 (17.4)	53 (18.4)
PPI	151 (35.6)	144 (35.6)	114 (33.5)	106 (32.6)	109 (35.2)	99 (34.4)
THoR	48 (11.3)	47 (11.6)	40 (11.8)	39 (12.0)	36 (11.6)	31 (10.8)
HoR	1 (0.2)	1 (0.2)	1 (0.3)	1 (0.3)	1 (0.3)	1 (0.3)
Drug for ED	8 (1.9)	8 (2.0)	5 (1.5)	5 (1.5)	4 (1.3)	3 (1.0)
*Patients with stable CAD and angina*						
*N*	*46*	*35*	*25*	*14*	*16*	*7*
Aspirin	43 (93.5)	28 (80.0)	20 (80.0)	13 (92.9)	13 (81.2)	5 (71.4)
Thienopyridines	4 (8.7)	6 (17.1)	7 (28.0)	1 (7.1)	2 (12.5)	0 (0.0)
Other antiplatelet agent	2 (4.3)	2 (5.7)	1 (4.0)	1 (7.1)	1 (6.2)	1 (14.3)
Oral anticoagulant	5 (10.9)	5 (14.3)	4 (16.0)	3 (21.4)	3 (18.8)	0 (0.0)
Beta-blocker	35 (76.1)	18 (51.4)	20 (80.0)	11 (78.6)	13 (81.2)	5 (71.4)
Ivabradine	11 (23.9)	8 (22.9)	2 (8.0)	1 (7.1)	0 (0.0)	0 (0.0)
Calcium antagonists	14 (30.4)	11 (31.4)	4 (16.0)	4 (28.6)	3 (18.8)	2 (28.6)
Verapamil or dilitiazem	4 (8.7)	5 (14.3)	2 (8.0)	1 (7.1)	1 (6.2)	0 (0.0)
ACE inhibitors	18 (39.1)	14 (40.0)	11 (44.0)	6 (42.9)	5 (31.2)	3 (42.9)
AT II receptor-blocker	15 (32.6)	14 (40.0)	7 (28.0)	7 (50.0)	7 (43.8)	1 (14.3)
LLD	42 (91.3)	32 (91.4)	23 (92.0)	14 (100.0)	15 (93.8)	7 (100.0)
Statins (if LLD)	36 (85.7)	25 (78.1)	17 (73.9)	7 (50.0)	10 (66.7)	4 (57.1)
Other antianginal agent	15 (32.6)	12 (34.3)	9 (36.0)	8 (57.1)	7 (43.8)	3 (42.9)
Trimetazine	0 (0.0)	0 (0.0)	0 (0.0)	0 (0.0)	0 (0.0)	0 (0.0)
Ranolazine	0 (0.0)	1 (2.9)	2 (8.0)	3 (21.4)	1 (6.2)	1 (14.3)
Diuretics	14 (30.4)	16 (45.7)	12 (48.0)	9 (64.3)	9 (56.2)	3 (42.9)
Other hypertensive agent	7 (15.2)	8 (22.9)	4 (16.0)	4 (28.6)	3 (18.8)	1 (14.3)
Digoxin and derivates	3 (6.5)	2 (5.7)	2 (8.0)	1 (7.1)	1 (6.2)	0 (0.0)
Amiodarone/dronedarone	2 (4.3)	3 (8.6)	1 (4.0)	0 (0.0)	0 (0.0)	1 (14.3)
Other antiarrhythmics	0 (0.0)	0 (0.0)	2 (8.0)	0 (0.0)	0 (0.0)	0 (0.0)
Other medication						
NSAIDs	11 (23.9)	6 (17.1)	3 (12.0)	2 (14.3)	1 (6.2)	1 (20.0)
Antidiabetic agents	15 (32.6)	5 (14.3)	3 (12.0)	1 (7.1)	2 (12.5)	0 (0.0)
PPI	21 (45.7)	15 (42.9)	9 (36.0)	6 (42.9)	7 (43.8)	3 (60.0)
THoR	6 (13.0)	5 (14.3)	4 (16.0)	2 (14.3)	1 (6.2)	0 (0.0)
HoR	0 (0.0)	0 (0.0)	0 (0.0)	0 (0.0)	0 (0.0)	0 (0.0)
Drug for ED	1 (2.2)	1 (2.9)	1 (4.0)	0 (0.0)	0 (0.0)	0 (0.0)

*ACE* angiotensin-converting enzyme, *AT* angiotensin, *CAD* coronary artery disease, *ED* erectile dysfunction, *HoR* hormone replacement therapy, *LLD* lipid-lowering drug, *N* number of patients, *NSAIDs* non-steroidal anti-inflammatory drugs, *PPI* proton pump inhibitor, *THoR* thyroid hormone replacement therapy

^a^percentages are of the data available

The primary treatment of patients with angina at baseline did not differ from the treatments of the whole Austrian patient population. Most of them received LLD (91.3%, statins: 85.7%), aspirin (93.5%), beta-blockers (76.1%) and other antianginal agents (32.6%); however, ivabradine, calcium antagonists, verapamil or dilitiazem, other antihypertensive agents as well as digoxin and derivates were more frequently used in patients with angina. The ACE inhibitors and diuretics were less frequently used. In addition, 45.7% of patients with anginal symptoms were treated with PPI, 32.6% with antidiabetic agents and 23.9% with NSAIDs. Throughout the follow-up period, treatments with aspirin, calcium antagonists and ivabradine saw a declining trend, whilst use of diuretics saw an increasing trend. The proportion of patients who were treated with LLD was very stable, but treatment with statins decreased. In addition, there was also a decline in the use of antidiabetic agents as well as thyroid hormone replacement therapies (Table 2).

Analysis of patient status over 5 years of CLARIFY follow-up showed no changes in mean body weight and mean BMI as well as no changes in systolic and diastolic blood pressure as well as heart rate. The prevalence of overweight, obesity and elevated LDL cholesterol plasma levels declined, but there was an increase in the proportion of patients with low HDL cholesterol plasma levels. Overall the proportion of smoking patients decreased, and only 1.9% of patients who did not smoke at baseline were current smokers in the 5th year (Table 3).

**Table 3:** Patient characteristics of Austrian patients with stable CAD and Austrian patients with stable CAD and angina during observation^i^

Parameter	1st year	2nd year	3rd year	4th year	5th year
*Patients with stable CAD (N [%])*					
*N*	*411*	*351*	*337*	*323*	*305*
Weight [kg]	81.6 (13.8)	81.9 (13.5)	82.1 (14.7)	81.9 (13.5)	81.4 (14.0)
BMI, mean (SD) [kg/m^2^]	27.7 (4.0)	27.9 (4.0)	27.9 (4.3)	27.7 (3.9)	27.6 (3.9)
HR (palpation), mean (SD) [bpm]	66.7 (9.6)	66.2 (9.9)	66.4 (11.3)	66.4 (10.3)	67.3 (11.3)
HR (ECG), mean (SD) [bpm]	66.0 (10.0)	65.8 (9.8)	66.1 (11.5)	66.5 (11.3)	67.0 (11.0)
SBP, mean (SD), [mm Hg]	133.4 (17.4)	132.9 (18.8)	133.3 (17.1)	134.3 (17.9)	133.7 (16.8)
DBP mean (SD), [mm Hg]	79.3 (9.3)	78.5 (9.5)	78.8 (9.4)	78.9 (9.7)	78.4 (9.4)
Waist circumference, mean (SD), [cm]	99.3 (12.0)	98.6 (11.5)	99.7 (13.1)	99.4 (11.2)	100.1 (13.6)
Overweight (%)^a^	305 (76.2)	270 (78.3)	253 (76.0)	232 (75.6)	224 (75.7)
Obesity (%)^b^	98 (24.5)	85 (24.6)	78 (23.4)	74 (24.1)	67 (22.6)
Smoking status (%)					
Current	45 (11.0)	35 (10.1)	33 (9.9)	29 (9.1)	27 (9.1)
Former	189 (46.3)	157 (45.2)	153 (45.7)	135 (42.5)	114 (38.3)
Never	174 (42.6)	155 (44.7)	149 (44.5)	154 (48.4)	157 (52.7)
Raised blood pressure (%)^c^	163 (40.8)	125 (36.2)	120 (36.0)	129 (42.0)	117 (39.5)
Raised LDL cholesterol 1 (%)^d^	120 (36.4)	96 (34.9)	86 (34.1)	72 (30.0)	69 (30.5)
Raised LDL cholesterol 2 (%)^e^	274 (83.0)	225 (81.8)	206 (81.7)	193 (80.4)	173 (76.5)
Lowered HDL cholesterol (%)^f^	58 (16.6)	48 (16.8)	52 (19.3)	53 (21.2)	55 (23.3)
Raised HbA1c (%)^g^	33 (28.0)	22 (23.2)	32 (35.6)	30 (36.1)	24 (31.2)
Smoking initiation (%)^h^	6 (1.7)	4 (1.3)	5 (1.7)	4 (1.4)	5 (1.9)
*Patients with stable CAD and angina*					
*N (%)*	*35 (8.6)*	*25 (7.2)*	*14 (4.2)*	*16 (5.0)*	*7 (2.3)*
Weight [kg]	79.5 (15.3)	81.9 (13.0)	79.7 (12.9)	76.9 (12.1)	71.7 (15.3)
BMI, mean (SD) [kg/m^2^]	26.9 (4.1)	27.1 (3.5)	27.2 (3.0)	27.6 (4.3)	24.0 (4.4)
HR (palpation), mean (SD) [bpm]	69.5 (11.4)	65.3 (12.0)	71.1 (16.9)	63.8 (9.0)	62.3 (4.8)
HR (ECG), mean (SD) [bpm]	68.5 (10.2)	65.1 (13.5)	68.4 (16.3)	63.9 (9.3)	63.3 (6.4)
HR ≥ 70 bpm (palpation)	17 (48.6)	5 (20.0)	6 (42.9)	3 (18.8)	1 (14.3)
SBP, mean (SD), [mm Hg]	129.8 (16.2)	131.8 (22.3)	140.0 (20.2)	136.8 (20.7)	138.0 (19.1)
DBP mean (SD), [mm Hg]	77.2 (10.3)	78.7 (8.4)	79.7 (13.5)	72.8 (11.3)	81.0 (14.8)
Waist circumference, mean (SD), [cm]	97.9 (13.5)	101.0 (11.7)	99.6 (11.6)	99.7 (15.1)	94.7 (12.9)
Overweight (%)^a^	24 (68.6)	19 (79.2)	11 (78.6)	10 (66.7)	3 (50.0)
Obesity (%)^b^	9 (25.7)	4 (16.7)	2 (14.3)	5 (33.3)	1 (16.7)
Smoking status *N* (%)					
Current	5 (14.3)	2 (8.0)	1 (7.1)	2 (12.5)	1 (14.3)
Former	14 (40.0)	13 (52.0)	6 (42.9)	5 (31.2)	3 (42.9)
Never	16 (45.7)	10 (40.0)	7 (50.0)	9 (56.2)	3 (42.9)
Raised blood pressure (%)^c^	9 (25.7)	8 (33.3)	7 (50.0)	8 (53.3)	3 (50.0)
Raised LDL cholesterol 1 (%)^d^	14 (50.0)	6 (30.0)	3 (25.0)	3 (25.0)	2 (50.0)
Raised LDL cholesterol 2 (%)^e^	25 (89.3)	16 (80.0)	9 (75.0)	8 (66.7)	4 (100.0)
Lowered HDL cholesterol (%)^f^	5 (16.1)	5 (23.8)	3 (25.0)	4 (28.6)	1 (25.0)
Raised HbA1c (%)^g^	3 (30.0)	2 (25.0)	2 (66.7)	0 (0.0)	0 (0.0)
Started smoking (%)^h^	0 (0.0)	0 (0.0)	0 (0.0)	0 (0.0)	1 (14.3)

*BMI* Body mass index, *bpm* beats per minute, *CAD* coronary artery disease, *DBP* diastolic blood pressure, *HbA1c* glycated hemoglobin, *HDL* high-density lipoprotein, *HR* heart rate, *LDL* low-density lipoprotein, *N* number of patients, *SBP* systolic blood pressure, *SD* standard deviation

^a^Defined as BMI ≥ 25–29.99 kg/m^2^

^b^Defined as BMI ≥ 30 kg/m^2^

^c^Defined as systolic blood pressure ≥140 mm Hg and diastolic blood pressure ≥90 mm Hg

^d^Defined as LDL cholesterol plasma level ≥1 g/l or 2.6 mmol/l

^e^Defined as LDL cholesterol plasma level ≥0.7 g/l or 1.8 mmol/l

^f^Defined as HDL cholesterol plasma level ≤40 mg/dl or 1.0 mmol/l

^g^in diabetic patients, defined as HbA1c level ≥7.0%

^h^for former/never smokers at baseline

^i^mean values as well as percentages are of the data available

The proportion of patients with angina decreased from 8.6% after the 1st year to 2.3% in the 5‑year follow-up. In addition, these patients showed a decrease in mean body weight and mean BMI. Furthermore, there was a decline in mean heart rate, and a heart rate ≥70 bpm was less common. In contrast, blood pressure increased and elevated blood pressure was more common in year 5 of CLARIFY compared to the year 1 assessment. The LDL cholesterol plasma levels did not change and the proportion of patients with lower HDL cholesterol plasma levels was growing, similar to the total Austrian patient population (Table 3).

At the end of the observation period, blood pressure was normalized in 58.5% of outpatients with stable CAD and treated hypertension, which was defined as SBP <140 mm Hg and DBP <90 mm Hg. Of the patients 70.4% had LDL cholesterol plasma levels below 1.0 g/l (2.6 mmol/l) and 24.5% had LDL cholesterol plasma levels <0.7 g/l (1.8 mmol/l), 42.1% of current smokers at baseline had stopped smoking during CLARIFY, 42.9% of patients with anginal symptoms achieved a heart rate ≤60 bpm and 26.0% of diabetic patients could reduce their HbA1c levels below 6.5% (Table 4).

**Table 4:** Treatment targets that were met 5 years after study inclusion in Austrian patients with stable CAD (*N*, [%])^h^

Variable	Patients with stable CAD (*N* = 305)
Normalized blood pressure^a^	134/229 (58.5)
Lowered LDL cholesterol 1^b^	138/196 (70.4)
Lowered LDL cholesterol 2^c^	48/196 (24.5)
Lowered HbA1c 1^d^	33/77 (42.9)
Lowered HbA1c 2^e^	20/77 (26.0)
HR ≤ 60 bpm^f^	3/7 (42.9)
Smoking cessation^g^	16/38 (42.1)

*bpm* beats per minute, *HbA1c* glycated hemoglobin, *HR* heart rate, *LDL* low-density lipoprotein, *N* number of patients

^a^in treated hypertensive patients, defined as systolic blood pressure <140 mm Hg, diastolic blood pressure <90 mm Hg

^b^in patients with dyslipidaemia at baseline, defined as LDL cholesterol plasma level <1.0 g/l or 2.6 mmol/l

^c^in patients with dyslipidaemia at baseline, defined as LDL cholesterol plasma level <0.7 g/l or 1.8 mmol/l

^d^in diabetic patients, defined as HbAc1 level <7.0%

^e^in diabetic patients, defined as HbAc1 level <6.5%

^f^in patients with anginal symptoms

^g^for current smokers at baseline

^h^percentages are of the data available

The primary outcome, defined as the composite of cardiovascular death, MI or stroke over 5 years occurred in 7.1% of Austrian patients and 8.7% of patients from the rest of the CLARIFY population. Cardiovascular death was observed in 3.5% of Austrian patients and 5.0% of patients in the rest of CLARIFY, all-cause death in 5.9% and 7.9% of each population, respectively and hospitalization for heart failure in 3.5% and 5.1%. The frequency of MI (fatal or non-fatal) and stroke (fatal or non fatal) was very similar between the populations (MI was observed in 3.3% and 3.4% of each population and stroke was observed in 1.9% and 2.1% of each population) (Table 5).

**Table 5:** Clinical events in Austrian patients and all CLARIFY patients^a^ with stable CAD (*N*, [%])^c^

Event	Austrian patients with stable CAD (*N* = 424)	CLARIFY patients with stable CAD (*N* = 31954)
All cause death	25 (5.9)	2519 (7.9)
Cardiovascular death	15 (3.5)	1604 (5.0)
Non-cardiovascular death	10 (2.4)	915 (2.9)
MI (fatal or non-fatal)	14 (3.3)	1092 (3.4)
Stroke (fatal or non-fatal)	8 (1.9)	678 (2.1)
Cardiovascular death or non-fatal MI	24 (5.7)	2329 (7.3)
Cardiovascular death, non-fatal MI or non-fatal stroke	30 (7.1)	2777 (8.7)
Non-fatal MI	9 (2.1)	786 (2.5)
Non-fatal stroke	6 (1.4)	525 (1.6)
Unstable angina	34 (8.0)	3405 (10.7)
Major bleeding	9 (2.1)	437 (1.4)
Coronary angiography	67 (15.8)	4562 (14.3)
PCI	34 (8.0)	2106 (6.6)
CABG	6 (1.4)	429 (1.3)
Revascularization (PCI or CABG)	39 (9.2)	2487 (7.8)
TIA	5 (1.2)	823 (2.6)
Hospitalization for CHF	15 (3.5)	1632 (5.1)
Cardiovascular death, non-fatal MI, non-fatal stroke or revascularization	30 (7.1)	2777 (8.7)
MI (fatal or non-fatal) or revascularization	42 (9.9)	3068 (9.6)
MI (fatal or non-fatal) or revascularization or unstable angina	56 (13.2)	5097 (16.0)
Cardiovascular hospitalization	124 (29.2)	9713 (30.4)

*CABG* coronary artery bypass graft, *CHF* congestive heart failure, *MI* myocardial infarction, *N* number of patients, *PCI* percutaneous coronary intervention, *TIA* transient ischemic attack

^a^complete CLARIFY population excluding Austria

^b^percentages are of the data available

European outpatients with CAD had a slightly lower heart rate than Austrian patients. The same could be found in patients with anginal symptoms at baseline but during the years of CLARIFY, the heart rate of Austrian patients with angina showed a stronger decline than the heart rate of patients with angina in the rest of Europe. In addition, the proportion of patients with heart rates above 70 bpm was lower among Austrian patients with angina at the end of documentation and relatively more Austrian patients with angina achieved a heart rate below 60 bpm. With respect to therapeutic heart rate-modulators, it could be seen that Austrian patients as well as patients from other European countries were primarily treated with beta-blockers. Differences could be found in the usage of ivabradine. Patients from other European countries were more frequently treated with ivabradine than Austrian patients, especially patients with angina. Verapamil or dilitiazem as well as digoxin and derivates played only a minor role in both (Table 6).

**Table 6:** Heart rate and heart rate-modulating medications in European^a^ patients with stable CAD and European^a^ patients with stable CAD and angina during observation^b^

Parameter	Baseline	1st year	2nd year	3rd year	4th year	5th year
*Patients with stable CAD (N, [%])*						
	*17,902*	*17,038*	*16,089*	*14,851*	*13,289*	*11,954*
HR, mean (SD) palpation (bpm)	67.3 (10.5)	66.3 (9.8)	65.9 (9.7)	66.0 (9.6)	66.1 (9.5)	66.0 (9.1)
HR, mean (SD) ECG (bpm)	66.5 (11.2)	65.7 (10.4)	65.3 (10.3)	65.4 (10.2)	65.6 (10.2)	65.5 (9.8)
Medications (*N*, %)						
Beta-blocker	14,077 (78.7)	12,900 (76.5)	11,917 (75.9)	10,910 (75.9)	9647 (76.5)	8568 (76.7)
Ivabradine	2852 (15.9)	3182 (18.9)	3289 (20.9)	3210 (22.3)	2965 (23.5)	2695 (24.1)
Verapamil or dilitiazem	914 (5.1)	807 (4.8)	728 (4.6)	603 (4.2)	472 (3.7)	407 (3.6)
Digoxin and derivates	381 (2.1)	374 (2.2)	347 (2.2)	338 (2.3)	292 (2.3)	262 (2.3)
*Patients with stable CAD and angina*						
*(N, [%])*	*4873 (27.2)*	*3599 (21.3)*	*3271 (20.6)*	*2929 (19.9)*	*2613 (19.9)*	*2384 (20.3)*
HR, mean (SD) palpation (bpm)	69.7 (10.8)	68.2 (9.5)	67.4 (9.3)	67.3 (8.9)	66.9 (8.8)	66.9 (8.3)
HR, mean (SD) ECG (bpm)	68.8 (11.6)	67.3 (10.2)	66.5 (9.9)	66.2 (9.6)	66.1 (9.6)	66.1 (8.9)
HR ≥ 70 bpm palpation (*N*, %)	2351 (48.2)	1495 (41.5)	1218 (37.2)	1086 (37.1)	866 (33.1)	755 (31.7)
HR ≤ 60 bpm palpation (*N*, %)	1102 (22.6)	860 (23.9)	827 (25.3)	745 (25.4)	696 (26.6)	570 (23.9)
Medications (*N*, %)						
Beta-blocker	3960 (81.3)	2944 (81.8)	2652 (81.5)	2381 (82.0)	2123 (82.4)	1917 (81.9)
Ivabradine	1417 (29.1)	1142 (31.7)	1156 (35.5)	1110 (38.2)	1030 (40.0)	925 (39.5)
Verapamil or dilitiazem	251 (5.2)	156 (4.3)	135 (4.1)	107 (3.7)	76 (3.0)	81 (3.5)
Digoxin and derivates	102 (2.1)	89 (2.5)	89 (2.7)	78 (2.7)	63 (2.4)	55 (2.4)

*bpm* beats per minute, *HR* heart rate, *N* number of patients, *SD* standard deviation

^a^Europe = Belgium, Bulgaria, Czech Republic, Denmark, France, Germany, Greece, Hungary, Ireland, Italy, Latvia, Lithuania, Luxembourg Netherlands, Poland, Portugal, Romania, Slovakia, Slovenia, Spain, Switzerland, United Kingdom, Ukraine

^b^mean values as well as percentages are of the data available

## Discussion

This analysis describes disease management and clinical outcome of Austrian outpatients with stable CAD as well as risk factors and life style management over 5 years of the CLARIFY registry. Main objectives of CAD management are to reduce morbidity and mortality and improve quality of life. Life style interventions, such as smoking cessation, reduction of body weight, increase in physical activities, control of blood pressure, cholesterol plasma levels and diabetes as well as the use of selective medications reduce the risk of cardiovascular events and improve survival [14, 15]. The aims of pharmacological management of stable CAD patients are to reduce angina and prevent cardiovascular events [17] and three classes of medication are shown to be essential in the treatment of CAD: lipid-lowering agents, antihypertensive agents and antiplatelet agents [18]. Risk factors found in Austrian outpatients with stable CAD were smoking, overweight, hypertension, raised LDL cholesterol plasma levels, elevated heart rate and a low level of physical activity [16, 18]. Anginal symptoms were more common in women than in men, which is consistent with the data of the whole CLARIFY population as well as the Euro Heart Survey [16, 20, 21]. It was shown that women present with angina twice as often as men [17]. Moreover, angina is the most frequent manifestation of CAD in women [22]. Interestingly, Austrian patients with angina were older than patients without angina, which contrasts with the results of the analysis of the international CLARIFY population where patients with angina were slightly younger than patients without angina. This could be the result of the small sample size of Austrian patients with angina compared to the patients with anginal symptoms in the total CLARIFY registry [6]. During the 5 years of observation there were life style improvements but recommended goals were not achieved by the majority of patients. It is known that smoking cessation has a positive effect on the prognosis of coronary disease [14, 23–25] and may be the most effective of all preventive measures. Smoking cessation is associated with a 36% reduction of mortality after MI [14], 13.2% of patients were current smokers at baseline, after 5 years 42.1% of these patients had stopped smoking. Another strong risk factor for cardiovascular complications is diabetes mellitus [14, 19, 26]. In the present study, diabetes had been previously diagnosed in 28.5% of all Austrian patients with CAD and in 41.3% of patients with angina. Current guidelines recommend disease management with HbAc1 below 7.0% [17]. While most diabetic patients had an HbAc1 level below 7.0% at baseline, the proportion of patients with HbAc1 > 7.0% remained constant during the observation period with less than half of those patients achieving treatment goals at 5 years. Overweight and obesity are risk factors for an increase in all-cause mortality in general and cardiovascular mortality in particular, because both have an adverse effect on blood pressure, dyslipidemia and glucose metabolism [14, 17, 23]. In the present analyses, overweight was present in approximately three quarters of patients over the whole observation period and the presence of obesity only slightly decreased from 23.3% at baseline to 22.6% at 5 years. Mean body weight, BMI as well as waist circumferences did not change. Dyslipidemia was present in the vast majority of Austrian patients with stable CAD. It is recommended that dyslipidemia should be managed according to the lipid guidelines with pharmacological aid and life style interventions. Investigations have shown that intensive LDL cholesterol lowering reduces the risk of cardiovascular events in patients with CAD. Moreover, it is associated with reduction in plaque volume [27–29]. Treatment goals of current guidelines are LDL cholesterol plasma levels below 1.8 mmol/l (<0.7 g/l) or a more than 50% reduction of LDL cholesterol plasma levels when the target level cannot be achieved [17]. More than 90% of Austrian patients were treated with LLD, which is clearly more than found in EUROASPIRE II or the Euro Heart Survey [14, 15]. Although LLDs were widely used, target LDL cholesterol levels <1.8 mmol/l were achieved by only 24.5% of patients. Nevertheless, 70.4% of patients could lower the LDL cholesterol plasma level below 2.6 mmol/l. A meta-analysis demonstrated that LDL cholesterol lowering by 40 mg/dl (1.04 mmol/l) resulted in a 23% decrease in CAD mortality [18]; therefore, lipid control is an unmet need that may be improved by the use of the novel proprotein convertase subtilisin/kexin type 9 (PCSK9) inhibitors alirocumab and evolocumab.

Elevated blood pressure is a major risk factor for CAD, heart failure and cerebrovascular disease [20]. Furthermore, high blood pressure following myocardial infarction is associated with an increased risk of reinfarction, stroke and cardiovascular death [14, 30]. Almost half of the Austrian patients with stable CAD and half of the patients with angina had elevated blood pressure and were treated with antihypertensive agents. Among these patients, 58.5% had a blood pressure of <140/90 mm Hg at the end of the observation period, which is recommended as treatment goal [17]; however, more intensive lowering of blood pressure below a systolic level of 120 mm Hg is associated with both a decrease of cardiovascular events but also blood pressure-related adverse outcomes [31]. Analyses of the international CLARIFY cohort showed that an elevated blood pressure >140/90 mm Hg was associated with an increased risk of stroke but SBP <120 mm Hg and DBP <70 mm Hg were also associated with an increased risk of cardiovascular events [32]. Thus, there is still uncertainty about the optimal blood pressure target; however, taking the currently recommended treatment target of <140/90 mm Hg, hypertension is not controlled in more than 40% of Austrian patients with stable CAD despite the broad use of antihypertensive agents. A total of 69–79% of patients received beta-blockers as first-line antihypertensive agents and 40–45% were treated with ACE inhibitors. The ACE inhibitors are primarily used in patients with CAD following myocardial infarction, in diabetic patients and patients with left ventricular dysfunction but all other patients with CAD should also receive ACE inhibitors once beta-blocker therapy has been established [18, 33]. Of the patients 28–30% were treated with AT II receptor blockers and finally approximately 17% received calcium antagonists, which are mainly used if beta-blockers are not tolerated [34]. Heart rate is a primary determinant of myocardial ischemia [9, 10, 12]. Heart rate lowering drugs have clear anti-ischemic and anti-anginal effects in patients with CAD [35], justifying a recommended target heart rate of 55–60 bpm [12]. This target was not achieved by the majority of Austrian patients. Moreover, and in contrast to patients in the rest of Europe, there was no reduction in mean heart rate over 5 years in Austrian patients with stable CAD despite use of beta-blockers in majority of patients; therefore, there is still much room for improvement in HR control in the management of stable CAD. In patients with angina and elevated HR addition of ivabradine is one option to reduce HR without an effect on blood pressure, thus achieving further antianginal and anti-ischemic efficacy. The INITIATIVE study showed that ivabradine is a potent anti-anginal agent and is as effective as atenolol in patients with angina [36]. Other investigations such as the ASSOCIATE study or the ADDITIONS study showed that ivabradine also improves symptoms and quality of life when combined with beta-blockers in patients with stable angina. Moreover, ivabradine produced additional anti-anginal and anti-ischemic benefits without untoward effects on tolerability [37–40]. Approximately one quarter of European CAD patients and almost 40% of European patients with angina received ivabradine as part of their CAD management. In contrast, only approximately 6% of Austrian patients with stable CAD received this medication. Even in the case of patients with anginal symptoms ivabradine was only rarely used. In years 4 and 5 of follow-up no patient with angina was treated with ivabradine; however, patient numbers were small, especially in years 4 and 5.

Antiplatelet therapy is an important component of CAD management [18]. Within the antiplatelet agents aspirin is the most important drug in the prevention of arterial thrombosis. This could also be seen in the present analysis. The benefit of the treatment with aspirin was shown in various studies and international guidelines [17, 36]. A meta-analysis showed that low-dose aspirin therapy (50–325 mg/day) is associated with a reduction of cardiovascular events and the risk of all-cause mortality but an important side effect is major bleeding [41]. Overall, 2.1% of Austrian patients were affected by major bleeding during the CLARIFY observation period, which corresponds to results in previous studies [41]. Several factors such as treatment duration, comedications or nonadherence to therapy have been shown to be associated with aspirin resistance [42]. Alternative agents are thienopyridine derivates such as clopidogrel, which act as antagonists of the platelet adenosine diphosphate receptor P2Y_12_. The benefit of clopidogrel compared with aspirin was shown in patients with previous myocardial infarction, stroke or peripheral vascular disease [43]. In Austria, thienopyridines were used in approximately 10% of patients. This is consistent with the recommendations of the European Society of Cardiology (ESC) proposing clopidogrel as second-line treatment, especially for aspirin-intolerant patients with CAD [17].

Revascularization was performed in 9.2% of patients, but most patients had a history of PCI or CABG. In patients with acute coronary syndromes revascularization reduces cardiovascular death and MI compared with conservative treatment [44, 45]; however, controversy exists over the benefit of revascularization in patients with chronic stable CAD compared with medical management [44, 46], and large studies are underway to settle this issue (ISCHEMIA trial, clinicaltrials.gov, NCT01471522). Results from the COURAGE study implicated that PCI did not reduce risk of death, MI and other cardiovascular events when added to current medical treatment in patients with stable CAD [47]. In contrast, a large meta-analysis resulted in a benefit of revascularization. The positive effects of revascularization are clearly shown in patients with anginal symptoms. In these cases, PCI or CABG more effectively relieve angina, reduce treatment with anti-anginal drugs as well as improve physical resilience and quality of life [48]. In this context, the ESC guidelines recommend revascularization on the basis of the presence of significant coronary artery stenosis, the severity of ischemia and the expected benefit for prognosis [17].

The primary outcome, defined as the composite of cardiovascular death, MI or stroke over 5 years occurred in 7.1% of patients, less frequently than in the rest of CLARIFY population (8.7%). The same could be found for cardiovascular death, all-cause death and hospitalization for heart failure, whereas the frequency of MI (fatal or non-fatal) and stroke (fatal or non-fatal) was very similar. The results are consistent with clinical trials such as COURAGE or CORONOR as well as the Euro Heart Survey which found an estimated annual mortality rate of 1.2–2.4%, an estimated annual incidence of cardiovascular death between 0.6% and 1.4% and an estimated annual incidence of non-fatal MI between 0.6% and 2.7% [47, 49, 50].

The Austrian CLARIFY cohort was relatively small, especially the patient population with anginal symptoms. Thus, we cannot exclude selection bias. In addition, symptoms and outcomes were based on reporting by the investigator, and not on standardized questionnaires or calibrated tests. Finally, more than 25% of patients were lost to follow-up. Thus, for a better understanding of the management and outcome of stable CAD in Austria studies with larger numbers of patients are needed.

## Conclusion

The characteristics of Austrian outpatients with stable CAD corresponded to those found in other developed countries and treatments followed the recommendations of the European guidelines. Thus, patients received LLD, aspirin, beta-blockers and ACE inhibitors; however, recommended goals of life style interventions including a heart rate less than 60 bpm and general risk factor management were not achieved by the majority of patients. Heart rate control and life style changes remain unmet needs of cardiovascular care in Austria.

## Appendix

### Full list of the CLARIFY investigators

Austrian CLARIFY investigators: Franz Michael Auhser, Andreas Beinhauer, Gerald Bode, Alexander Dzien, Michael Felbermayer, Michel Feldner-Busztin, Wolfgang Filip, Johannes Föchterle, Ursula Frank, Elisabeth Galehr, Walter Gritsch, Valery Hadjiivanov, Peter Haralambus, Rainer Herrmann, Thomas Honsig, Karl-Heinz Karner, Raimund Kaserbacher, Doris Kerö, Lucas Kleemann, Claus Lavicka, Mahr Andreas, Karl Mayr, Herbert Mayr, Regina Mika, Martin Georg Millauer, Beate Mörz-Proszowski, Ewald Moser, Peter Painsipp, Thomas Perger, Rudolf Raffelsberger, Roland Schwarz, Gabriele Sepp, Günter Steurer, Anton Suntinger, Ursula Teleky, Hildgard Topf, Joachim Toplak, Kurt Walcher, Erich Ziak.
